# A Step-by-Step Damage Identification Method Based on Frequency Response Function and Cross Signature Assurance Criterion

**DOI:** 10.3390/s21041029

**Published:** 2021-02-03

**Authors:** Jiawang Zhan, Fei Zhang, Mohammad Siahkouhi

**Affiliations:** School of Civil Engineering, Beijing Jiaotong University, Beijing 100044, China; 18115057@bjtu.edu.cn (F.Z.); mohammad.siahkouhi@gmail.com (M.S.)

**Keywords:** structural health monitoring, damage identification, frequency response function (FRF), model updating, simply supported beam, cross-signature assurance criterion (CSAC)

## Abstract

This paper aims to present a method for quantitative damage identification of a simply supported beam, which integrates the frequency response function (FRF) and model updating. The objective function is established using the cross-signature assurance criterion (CSAC) indices of the FRFs between the measurement points and the natural frequency. The CSAC index in the frequency range between the first two frequencies is found to be sensitive to damage. The proposed identification procedure is tried to identify the single and multiple damages. To verify the effectiveness of the method, numerical simulation and laboratory testing were conducted on some model steel beams with simulated damage by cross-cut sections, and the identification results were compared with the real ones. The analysis results show that the proposed damage evaluation method is insensitive to the systematic test errors and is able to locate and quantify the damage within the beam structures step by step.

## 1. Introduction

Bridges constitute a large portion of civil structures located on highways and railways. These structures should be frequently inspected for potential damage arising from overloading, environmental effects, fatigue, age, and dynamic impacts. Thus, developing new damage identification methods is necessary. Over the years, many researchers have developed various bridge structural health-monitoring methods [1,2,3]. Bridge health assessment is usually conducted using static and/or dynamic methods. However, dynamic methods are always preferred over static ones. The static methods are time-consuming and require complicated platforms and traffic blocking. Therefore, to assess a large number of bridges, it is better to use dynamic methods, especially from time and resource optimization perspectives. Thus, bridge structural damage assessment methods based on dynamic tests have become more and more popular [4].

There are many different types of dynamic methods available in the literature. These methods include but not limited to the following: using incomplete modal data by Bayesian approach and model reduction technique [5], a mode shape component-specific damage index [6], plots of variation of model parameters [7], frequency-based method versus mode-shape-based method [8], modal strain energy change method [9], the frequency-response function (FRF) curvature method [10], and other methods using vibration-based structural damage detection [11].

Natural frequencies, mode shapes, mode shape curvatures, modal strain energy, and other mode-based dynamic characteristic data are usually obtained from the measured FRF data, at dominant frequencies around the resonances. De Roeck et al. [12] used the finite element method (FEM) modeling to validate the model-based damage identification method. The component of dynamic methods that use FRF to identify damage is very effective in theory and has a proven historical record of detecting, locating, and quantifying the severity of the damage. In practical applications, however, it is difficult to measure high-order modes due to the limitation of vibration measurement technology. It has been concluded that the number of identification parameters is mostly limited by the order of the test modes and that the detection accuracy is influenced by the modal errors [13]. Mousavi et al. [14] located and quantified the bridge damage using an Artificial Neural Network based on complete ensemble empirical mode decomposition with adaptive noise technique. Kildashti et al. [15] monitored bridge health using a drive-by-bridge inspection method. It is concluded that the vehicle vibration response identified the bridge damage, and the damage location and its severity were also identified. However, this method needs more investigation in the case of vehicle parameters optimization because this method highly depends on certain vehicle parameters. Zhan et al. [16] discussed using a model updating method for box-beam bridges. In this research, the frequency responses of the bridge were obtained under passing vehicles, and the objective function was constructed, accordingly. The model was updated based on damage indices and the damage location and severity were detected.

In order to detect damage within the structure, particularly irregular shapes and complicated structures, a mechanism called “global vibration-based structural damage detection” [17,18] is proposed. This methodological approach is based on the principle that any structure can be taken as a dynamic system with stiffness, mass, and damping properties. Once there are some damages to the structure, the structural parameters will be changed, along with the FRF and modal parameters of that structural system. Thus, the change in the modal parameters can be theoretically considered as a sign of physical damage to the structure [19,20,21,22,23]. FRF reflects the inherent characteristics of the structure, which can easily be computed using the bridge acceleration spectrum analysis. Esfandiari et al. [24] proved that using FRF data, instead of modal data, has the benefit of avoiding analysis errors, especially when the extracted modes of the structure are close to each other. Dascotte et al. [25] proposed the similarity of the FRF in the pre-damage and post-damage frequency domain and tried to use the model modification method for damage identification. Lin et al. [26] corrected the model through amplification of the FRF to identify structural damage. Ting et al. [27] proposed a method to improve the FRF sensitivity. In general, the basic idea of these methods is to evaluate the amplitude of FRF by comparing the theoretical and the measured values. From these literature reviews [20,21,22,23,24,25,26,27,28], it can be inferred that FRF has the potential to be used in the detection of structural behavior and damage.

Using FRF is often accompanied by physical uncertainties due to inaccuracies in the measurement, difficulties in the field measurements, and human errors [28,29]. Model updating-based methods can therefore be explored to overcome some of these problems. Some studies that have successfully used model updating methods for damage identification based on FRF are discussed below.

Esfandiari [29] studied the model-updating method using FRF of incomplete strain data. A sensitivity-based algorithm was proposed for finite element model updating. It was used to select the measurement frequency points for model updating and evaluating the weighting of the sensitivity equations. Sipple et al. [30] assessed a FEM updating model using FRFs and numerical sensitivity analysis. Cheung et al. [31] proposed a hybrid Monte Carlo simulation (HMCS) that could be used to solve higher-dimensional Bayesian model updating problems. Lin et al. [26] investigated model updating of damped structures using FRF data. To solve the problem of complexity of the measured FRF and modal data, FRF data were used to identify the damping coefficients for the cases of proportional damping and general non-proportional damping. Imregun et al. [32] used FRF data to prove that consistent with many other updating techniques, the incompleteness of the experimental model remains a major obstacle while correcting the analytical model. The main challenge is that noise on the FRF data has an adverse effect on locating the errors. Additionally, complex FRF data with noise, either simulated or measured, make the convergence process very slow and often numerically unstable.

Various studies have been conducted to reduce these errors and the complexity of the FRF data. The complex measurement process, needing high accuracy and slow convergence process, inevitably calls for modifying this method (using FRF data) and subsequently, simplify the damage identification method. Considering the conditions referred above, this paper targets to improve the theory of model modification based on the modal parameters and FRF. An objective function is constructed using the cross-signature assurance criterion (CSAC) of the FRF between the adjacent measurement points and the natural frequency. The constraint conditions and identification parameters can then be effectively increased by increasing the number of measurement points. In the case of unknown damage location and limited accelerometer sensors, a step-by-step damage identification method is proposed. Thus, in this paper, a new step-by-step damage identification method based on FRF is proposed to identify the damage location and quantify the severity of damage in simply supported beams. This method of damage identification using CSAC can be used for other types of beams such as continuous beam, cantilever, etc., in addition to different load patterns such as concentrated, uniform, and multiplied loads. Once the FRF response can be captured, this method has the capability to be used, although it is expected that the chosen frequency band changes considering the different RFR shapes of the above-mentioned beams rather than simply supported beams. Simply supported beam in this study has been selected due to the number of simply supported deck bridges that exist in China.

## 2. Theory

The paper mainly adopts the method of model updating, the objective function is obtained using frequency and the CSAC of the FRF between the adjacent measurement points of the structure. This can reduce the influence of the vibration-related amplitude measurement error on the FRF, and subsequently, increase the number of constraint equations and identified variables. Consequently, the damage location and damage severity can be determined. The key step of the structural damage identification method is the updating of the finite element model, which includes three aspects, namely, cross-signature assurance criterion, damage index (DI), and objective function construction.

### 2.1. Cross-Signature Assurance Criterion

**Figure 1:**
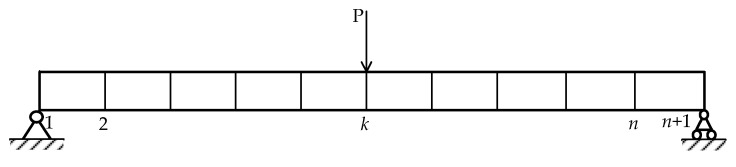
A simply supported beam under external loading.

The dynamic motion equation for a simply supported beam with *n* degrees of freedom (DOFs) under external loading is shown in Figure 1. The FRFs between the excitation impact force at point *p* and the measurement point *k* of the acceleration responses in the frequency domain are expressed as shown in Equation (1),

(1) $$H_{pk}^{a}\left(\omega \right)=\sum_{r=1}^{n}\frac{-\omega^{2}\phi_{pr}\phi_{kr}}{-M_{r}\omega^{2}+jC_{r}\omega +K_{r}}$$

where [*M_r_*], [*C_r_*], and [*K_r_*] are the *r*th (*r* = 1, …, n) modal mass, damping, and stiffness matrices, $\phi_{pr}$ and $\phi_{kr}$ are the *r*th regular vibration modes of the system at point *p* and *k*, and ω is the dominant frequency.

The CSAC reflects the shape similarity of the FRF between adjacent measurement points. Since the shape of an FRF is determined by the number of resonant peaks, the CSAC function is relative to changes in mass and stiffness values. So CSAC can be used to identify the change of structural stiffness. For example, the CSAC index between the adjacent measurement Points *p* and *q* under the excitation impact force *k* is calculated as follows [25]:

(2) $$\mathrm{CSAC}\left(p,q\right)=\frac{{\left(\left|{\left\{H_{pk}\right\}}^{*}\{H_{qk}\}\right|\right)}^{2}}{\left({\left\{H_{pk}\right\}}^{*}\{H_{pk}\})({\left\{H_{qk}\right\}}^{*}\{H_{qk}\}\right)}$$

where

(3) $${\left\{H_{pk}\right\}}^{*}=\left\{H_{pk}\left(\omega_{1}\right),\cdots ,H_{pk}\left(\omega_{l}\right),\cdots ,H_{pk}\left(\omega_{m}\right)\right\}$$

(4) $${\left\{H_{qk}\right\}}^{*}=\left\{H_{qk}\left(\omega_{1}\right),\cdots ,H_{qk}\left(\omega_{l}\right),\cdots ,H_{qk}\left(\omega_{m}\right)\right\}$$

where: $\omega_{l}\left(l=1,2,\ldots ,m\right)$ is the dominant frequency in the selected frequency range, and *m* is the number of frequency points. In this paper, it is assumed that the frequency band is between first and second natural frequencies. Detailed numerical analysis is presented in Section 3.2 of this paper.

Assuming that there are systematic scaling errors in measuring the beam vibration responses, then the whole amplitudes of FRF spectrums at measurement points *p* and *q* become *a* and *b* times that of the true value, respectively. Then, the contaminated CSAC index becomes

(5) $$\mathrm{CSAC}\left(p,q\right)=\frac{{\left(\left|{\left\{aH_{pk}\right\}}^{*}\{bH_{qk}\}\right|\right)}^{2}}{\left({\left\{aH_{pk}\right\}}^{*}\{aH_{pk}\})({\left\{bH_{qk}\right\}}^{*}\{bH_{qk}\}\right)}={\mathrm{CSAC}}^{0}\left(p,q\right)$$

From Equation (5), it can be seen that the CSAC index is not affected by the systematic scaling errors and is equal to the initial value. The results of the CSAC index calculation are related to the frequency range. Generally, Equation (5) is used for matching the measured and the theoretical FRF spectrum in a specific frequency range. The CSAC index is typically between 0 and 1. The limit value of zero (0) indicates that there is no similarity between the two FRFs, while the limit value (1) indicates that the two FRFs are identical.

### 2.2. Damage Index (DI) Formulation

Damage directly affects the stiffness of the structure. The stiffness change is usually simulated by reducing the elastic modulus *E* [33,34] while keeping the moment of inertia *I* of the structural section constant. Assuming that $E_{0j}$ is the initial elastic modulus of the *j*th element of the beam and $E_{dj}$ represents the elastic modulus of the element with damage, then the DI $\alpha_{j}\left(0\leq \alpha_{j}<1\right)$ of the *j*th element can be defined as follows:

(6) $$\alpha_{j}=1-\frac{E_{dj}I}{E_{0j}I}=1-\frac{E_{dj}}{E_{0j}}$$

where *j* = 1, 2, …, *n,* while *n* is the total number of bridge elements. The DI of each element can be used to describe the deterioration severity of a simply supported beam as follows:

(7) $$\alpha =\left\{\alpha_{1},\alpha_{2},\cdots ,\alpha_{n}\right\}$$

### 2.3. Objective Function Formulation

**Figure 2:**
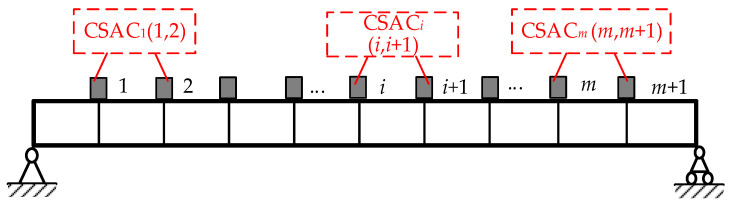
Schematic layout of cross-signature assurance criterion (CSAC) indices between adjacent points.

The objective function is usually defined as the summation of the square of residuals. In this paper, the objective function is formulated using the CSAC index vector residuals (δ(α)) and frequency residuals (φ(α)) as follows:

(8) $$F(\alpha )=\lambda_{\mathrm{CSAC}}\sum_{i=1}^{m}\delta_{i}^{2}\left(\alpha \right)+\sum_{j=1}^{2}\lambda_{j}\varphi_{j}^{2}\left(\alpha \right)$$

where δ(α) is the CSAC index vector residual between the adjacent measurement points in the beam as subsequently illustrated in Equation (9), and $\varphi_{j}\left(\alpha \right)=f_{j}^{d}-f_{j}^{0}$ is the difference between the *j*th measured frequency $f_{j}^{d}$ and the theoretically calculated frequency $f_{j}^{0}$ of the beam. Superscripts d and 0 are used to denote the measured and theoretical values, respectively. $\lambda_{\mathrm{CSAC}}$ and $\lambda_{j}$ are the weight coefficients of the CSAC index and the *j*th frequency, respectively. Figure 2 illustrates the schematic layout of the CSAC index between the adjacent measurement points. When the impact force (*P*) is applied to the beam, it is assumed that CSAC^0^ and CSAC^d^ are the theoretical and measured value between the measurement points, respectively, as illustrated in Equation (10),

(9) $$\delta_{i}\left(\alpha \right)={\mathrm{CSAC}}_{{}^{i}}^{d}(i,i+1)-{\mathrm{CSAC}}_{i}^{0}(i,i+1)$$

(10) $$\begin{array}{l} {\mathrm{CSAC}}^{d}=\left\{{\mathrm{CSAC}}_{{}_{1}}^{d}(1,2),\cdots ,{\mathrm{CSAC}}_{i}^{d}(i,i+1),\cdots ,{\mathrm{CSAC}}_{m}^{d}(m,m+1)\right\}\\ {\mathrm{CSAC}}^{0}=\left\{{\mathrm{CSAC}}_{1}^{0}(1,2),\cdots ,{\mathrm{CSAC}}_{i}^{0}(i,i+1),\cdots ,{\mathrm{CSAC}}_{m}^{0}(m,m+1)\right\} \end{array}$$

where ${\mathrm{CSAC}}_{i}^{d}(i,i+1)$ and ${\mathrm{CSAC}}_{i}^{0}(i,i+1)$ are the measured and theoretical CSAC indices calculated using Equation (2) between the measurement points *i* and *i* + 1, respectively.

Compared with the objective function from the literature, the current objective function has the following characteristics:

(1) The shape similarity of the FRF spectrum in the frequency domain can be directly used to reduce the influence of the systematic test errors in the specified frequency range;
(2) When the objective function is constructed using the CSAC index between the adjacent measurement points of the structures, the total number of constraint equations can be increased by increasing the number of measurement points. The model is thus modified to effectively accommodate the number of parameters. When *m* + 1 measurement points are arranged, the number of CSAC exponents between the adjacent points is *m*, which is much higher than that of the constraint equations constructed using low order modes.

### 2.4. Optimization and Convergence Criteria

After the DI is selected and the objective function assembled, further research needs to be conducted for optimization, which is primarily aimed at extracting the exact value of stiffness of the beam. In this paper, the Lsqonlin function in the MATLAB toolbox is used to solve the constrained nonlinear least-squares optimization problem. The objective function F(α) is minimized by optimizing the identification index vector **α**. The convergence criteria are set as follows:

(11) $$F(\alpha )<\mu ;\;\left|F(\alpha_{n-1})-F(\alpha_{n})\right|/F(\alpha_{n-1})<\varepsilon ;\;\alpha^{L}<\alpha <\alpha^{U};\;n<N_{\max}$$

where *μ* is the allowable residual, *n* is the number of iterations, *ε* is the allowable difference, **α**^L^ and **α**^U^ are the limits of the vector **α**, and *N*_max_ is the maximum number of iterations, respectively.

### 2.5. Damage Identification Process

**Figure 3:**
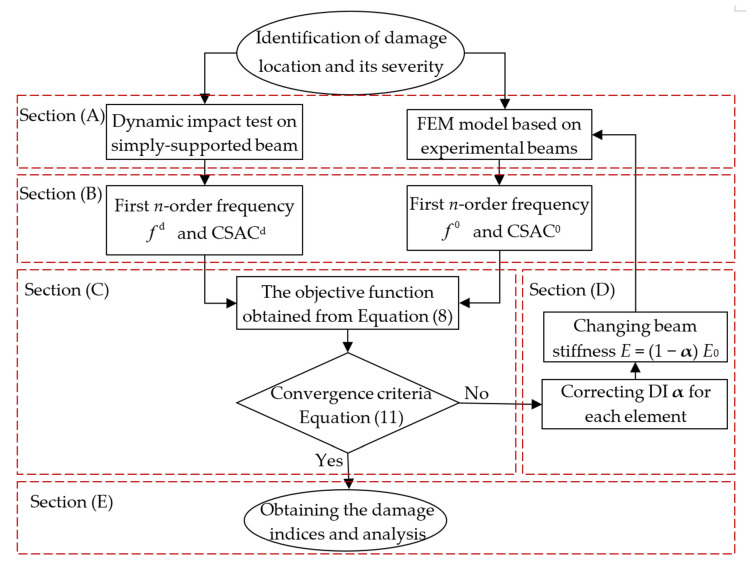
Flowchart of the step-by-step damage identification method.

Generally, identifying the location and severity of damage in bridges requires using some detection methods. In this paper, a damage identification process is executed based on the model updating and modified FRF-based objective function (see Figure 3). On the same basis, a step-by-step method is proposed for evaluating the bridge damage, with conditions of using the minimum number of available sensors. It should be mentioned that more sensors in the first step of damage detection will result in a narrower damage zone for the next steps and consequently sooner damage detection, however, with a minimum number of sensors also damage detection can be done. This is a method of evaluation accomplished through gradual measurements and refining elements. The damage identification process comprises of the following steps:

(1) Dividing the beam into sections and determining the sensor installation location. Thereafter, develop a FEM model of the beam for further analysis, which is denoted Section (A) in the flow chart in Figure 3.
(2) Measuring the dynamic responses of the bridge during experimentation and quantitative estimations using theoretical methods with the FEM model. Thereafter, calculating the FRF and the corresponding CSAC indices, which is denoted as Section (B) in the flow chart.
(3) Using the model updating method proposed in this paper to determine the DI for constructing the objective function, with respect to both the results obtained from the actual measurement and theoretical analysis, which is denoted as Section (C) in the flow chart.
(4) The measurement elements and sensors are arranged uniformly as done in Step 1, although, the damaged element is refined into smaller elements and more sensors are used in this zone. If the objective function does not converge, then the DI **α** will be modified and consequently, the structural stiffness will be changed. This step can be iteratively repeated to approach the actual damage, which is denoted as Section (D) in Figure 3.
(5) Correcting the finite element model and presenting the damage indices with respect to the analysis location and severity of the damage, as denoted in Section (E) of the flow chart in Figure 3.

## 3. Model Testing and Numerical Simulations

### 3.1. Beam Characteristics

**Figure 4:**
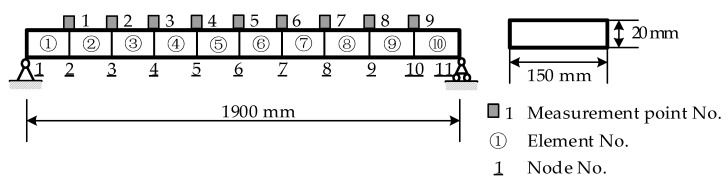
Finite element model of the simply supported steel beam.

The simply supported beam shown in Figure 4 is used for model demo and testing in this study. The span of the simply supported beam is 1900 mm. The thickness and width are 20 mm and 150 mm, respectively. The material of the beam is steel with an elastic modulus and density of 2.06 × 10^5^ MPa and 7850 kg/m^3^, respectively. The beam is evenly divided into 10 elemental structures and 11 nodes. To effectively stimulate the vibration of the bridge and reduce noise in the low-order mode of the simply supported beam, an impact load was applied on Node 8. The damage was simulated by reducing the stiffness of the beam elements using four damage case scenarios listed in Table 1. The first two natural frequencies of the intact beam are 12.87 Hz and 51.48 Hz, respectively.

### 3.2. Sensitivity Analysis of the CSAC Index Versus Frequency

**Figure 5:**
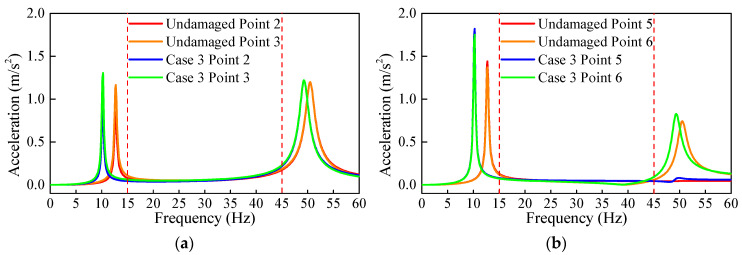
Frequency response function (FRF) spectrum comparison between undamaged and damaged conditions, Case 3; (**a**) measurement points 2, 3 and (**b**) measurement points 5, 6.

It can be seen in Equation (2) that the calculated results from the CSAC index rely on the frequency range. Thus, it is necessary to analyze the sensitivity of the CSAC index to the damage of the adjacent measurement points with different frequency ranges. The acceleration responses of the measurement points are recorded, and the FRFs for different damage cases are computed. From the FRF comparison between undamaged and damaged conditions, i.e., Case 3 shown in Figure 5, it can be seen from the FRF spectrum that the first two natural frequencies of the beam are reduced due to the damage. These first two mode shapes are sensitive to the damage; therefore, they are used to calculate CSAC. These are minimum modes that are needed for CSAC calculation, and if other modes can be detected sensitive to damage, they can be used as well. As shown in Table 2, four different frequency ranges near the first two frequencies are selected to calculate the CSAC. This is because, for most simply supported beams, the first two natural frequencies are relatively easy to measure.

**Figure 6:**
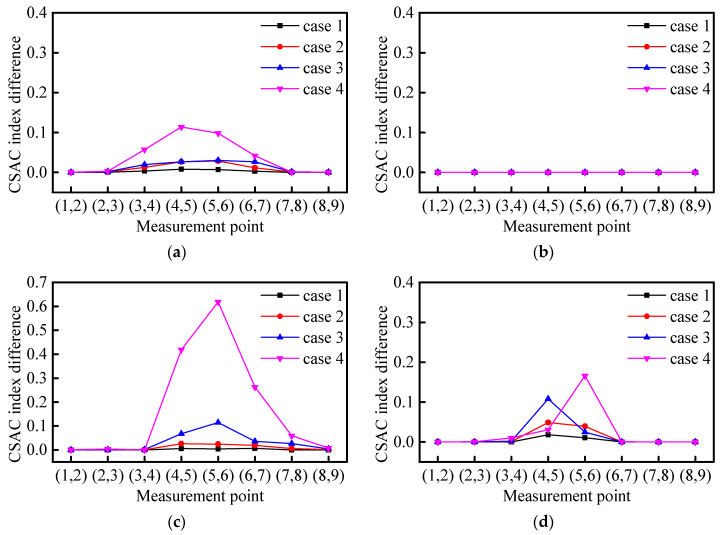
Comparison of damage cases with respect to different frequency ranges; (**a**) frequency range 1; (**b**) frequency range 2; (**c**) frequency range 3; and (**d**) frequency range 4.

It can be seen from Figure 5 that the FRF spectrum of the measurement points on both sides of the element without damage is highly consistent. At the same time, the FRF (δ(α)) are in different frequency ranges. Figure 6a shows that the calculated δ(α) using the frequency range including the first two frequencies will change with the occurrence of damage, but this change is very small and does not have a positive correlation with damage. 

Figure 6b shows δ(α), calculated using the frequency range including the first frequency, which is almost zero (0). This is because the FRF amplitude has a maximum value at the first natural frequency. When the CSAC in this frequency range is calculated, the amplitude at the predominant natural frequency occupies a high proportion, i.e., a condition where the CSAC values approach to 1. Therefore, δ(α) values are small in the nominated frequency range. Figure 6c shows the calculated δ(α) using the frequency range between the first and second natural frequencies will increase with the damage. Because there is no first-order frequency, the calculation result of this frequency range will not be affected by the high similarity of the first-order frequency. 

Figure 6d shows the calculated δ(α) using the frequency range including the second-order frequency will change with damage, but it is mainly occurring in the mid-span rather than on both sides of the damaged element. The amplitude of the second mode shape at the mid-span is close to zero (0), thus the δ(α) between measurement points near the mid-span is much bigger, which will cover the real difference due to damage. 

Based on the above-mentioned analysis, the frequency range between the first and second natural frequencies is chosen to calculate the CSAC indices between the measurement points.

### 3.3. Quantitative Analysis of the Beam Damage

**Figure 7:**
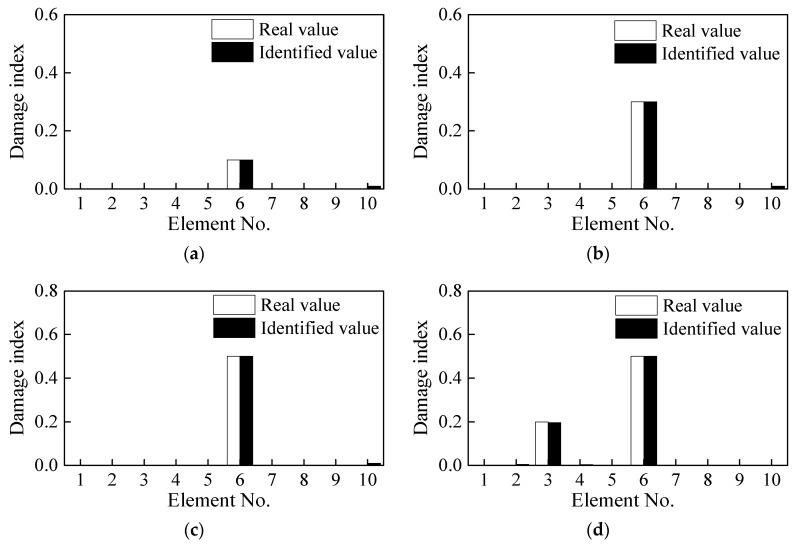
Damage identification results under different cases; (**a**) Case 1, (**b**) Case 2, (**c**) Case 3, and (**d**) Case 4.

**Figure 8:**
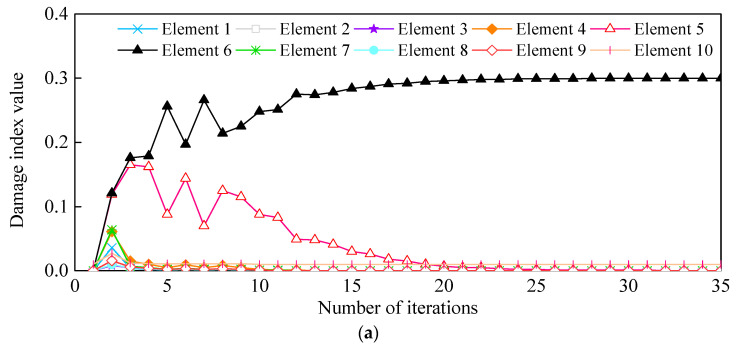

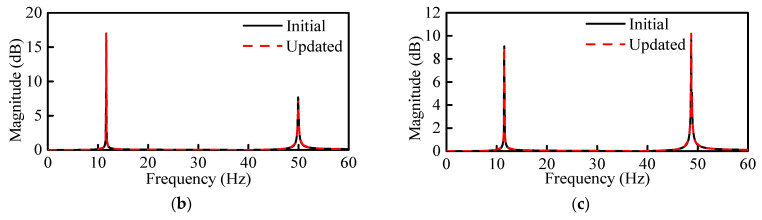
(**a**) Iteration numbers of Case 2 for the convergence and comparison of the acceleration FRF curves before and after model updating; (**b**) Case 3, and (**c**) Case 4.

The CSAC index of the adjacent measurement points is calculated using the principle mentioned in Figure 3 and thereafter combined with the first two natural frequencies to construct the objective function. The CSAC index and the frequency of the adjacent measurement points in the damaged beam are taken as the “measured value” and then the finite element (FE) model is modified to identify the induced damage. As shown in Figure 7, both single and double damage occurring in Elements 3 and 6 are correctly identified. Although there is some unrealistic damage in some elements, which mainly comes from the model and iterative errors, their damage indices are negligibly much lower and can be ignored. As shown in Figure 8, the objective function converges after about 20 iterative steps and the DI of Element 6 is effectively/correctly identified.

### 3.4. Influence of Noise on Damage Identification

**Figure 9:**
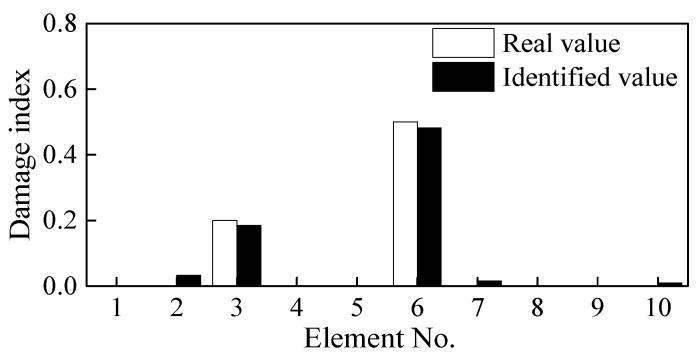
Identification results of double damage with 5% noise.

To analyze the influence of the test errors, a certain level of random noise is added to the test acceleration responses. The damage identification process is then conducted with the acceleration data after noise “pollution.” Using the acceleration response with 5% random noise, the damage for Case 4 is identified as shown in Figure 9. Although some unreal ignorable damage occurs at some elements under the influence of test noise, the damage at Elements 3 and 6 could still be accurately identified and quantified. This indicates that the damage identification method proposed in this paper has adequate robustness and noise immunity. Furthermore, the results suggest that synchronization identification of multiple damages can also be achieved. 

### 3.5. Step-by-Step Damage Identification Process

**Figure 10:**
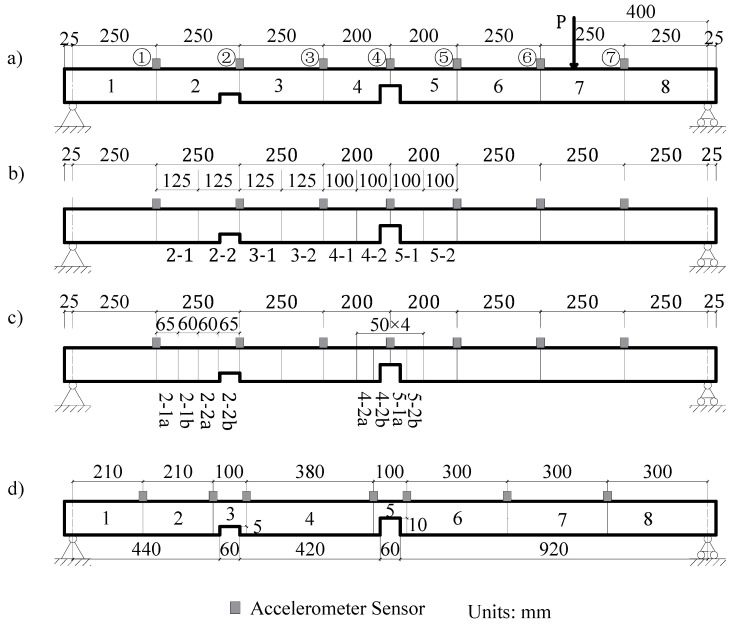
The first layout of the load, beam elements, and sensors (**a**) with a gradual subdivision of elements as (**b**–**d**), respectively.

The simply supported steel beam shown in Figure 4 is adopted on the assumption that damage occurred concurrently within 1/4 span and mid-span of the beam. Figure 10a shows these damage locations. The lengths of the damage of the two locations are both 60 mm, with DI values of 0.6 and 0.8, respectively. The first two frequencies of the damaged beam are 11.2 Hz and 48.7 Hz, respectively. The test equipment has only eight channels, with seven channels measuring the acceleration response of the measurement points, and one channel measuring the input impact force. The step-by-step method is used to identify and pinpoint the damage.

#### 3.5.1. Initial Identification of the Damage Location

**Figure 11:**
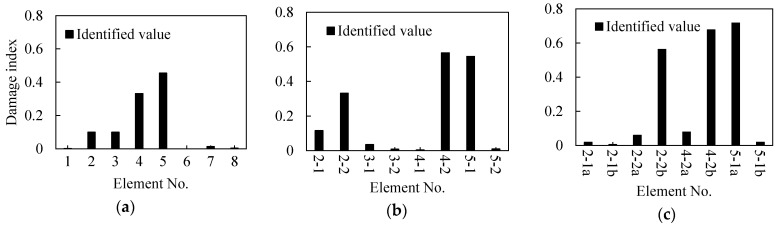
Identified values of damage in each element based on Figure 10; (**a**) damage indices of Figure 10a, (**b**) damage indices of Figure 10b, and (**c**) damage indices of Figure 10c.

As shown in Figure 10a, the beam is subdivided into eight elements, seven accelerometer sensors are mounted on the beam considered as measuring points, and an impact load is applied at 400 mm from the right support. Two damage with a width of 60 mm and depth of 5 mm (Elements 2 and 3) and 10 mm (Elements 4 and 5) are induced/marked on the beam. From the identification results shown in Figure 11a, it can be seen that the damage indices of Elements 2, 3, 4, and 5 are quantitatively much higher than other elements. 

#### 3.5.2. Damage Identification after Subdivision of the Elements

To confirm the location accuracy of damage, the initial identified Elements 2, 3, 4, 5 are each subdivided into two elements of equal lengths (Figure 10b). This is on the assumption that the other elements are undamaged. Thereafter, the damage indices of the Elements 2-1, 2-2, 3-1, 3-2, 4-1, 4-2, 5-1, and 5-2 are used as the identification variables and the model correction is repeated. The identification results shown in Figure 11b indicate that Elements 2-1, 2-2, 4-2, and 5-1 significantly have larger damage indices than other elements. These elements can be further be subdivided to locate the changes. As shown in Figure 10c, the identified damaged elements are further subdivided again on the assumption that the remaining elements are undamaged. The identification results after refining the elements are shown in Figure 11c. It is clear from the figure that the damage locations are Elements 2-2b, 4-2b, and 5-1a and that the damaged elements’ lengths are 65 mm, 50 mm, and 50 mm, respectively.

#### 3.5.3. Identification of Damage Location and Severity

**Figure 12:**
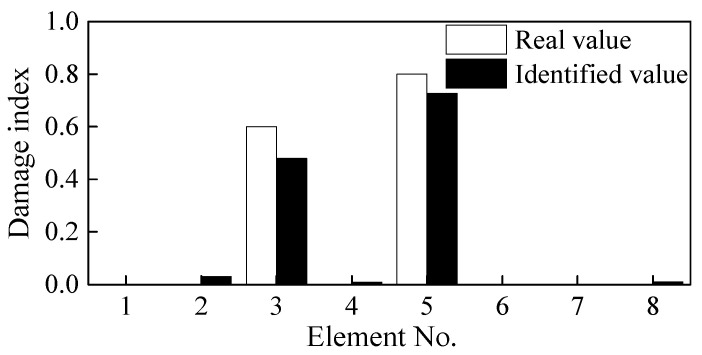
Damage identification results after adjusting of measurement points.

Because Elements 4-2b and 5-1a are adjacent, two sensors are arranged at both ends of the damaged elements at 100 mm apart to completely cover the identified damaged element. For ease of comparison, the distance between the two ends of Element 2-2b is also changed to 100 mm. As shown in Figure 10d, the locations of the other sensors are adjusted according to the location of Elements 3 and 5. The beam is accordingly divided into eight elements. The identified damage indices of the eight elements are shown in Figure 12.

Based on Figure 12, it can be easily determined that Elements 3 and 5 are damaged. The damage indices of these two elements are 0.48 and 0.73, respectively, which differs from the real values of 0.6 and 0.8. The lengths of the damaged elements are taken as 100 mm, which is larger than the real damage length of 60 mm. The identified DI is the average value along with the whole element, and thus, it is a little smaller than the actual value. These results from the above analysis demonstrate that the step-by-step identification method can be used simultaneously to locate and quantify damage in simply supported beams.

## 4. Laboratory Experimentation and Model Verification

### 4.1. Beam Fabrication

**Figure 13:**
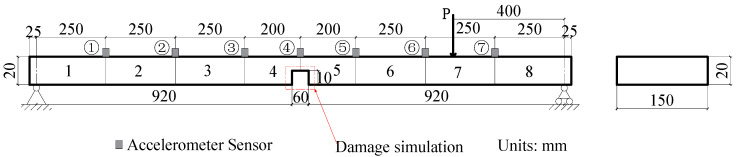
Schematic drawing of the simply supported steel beam (mm).

**Figure 14:**
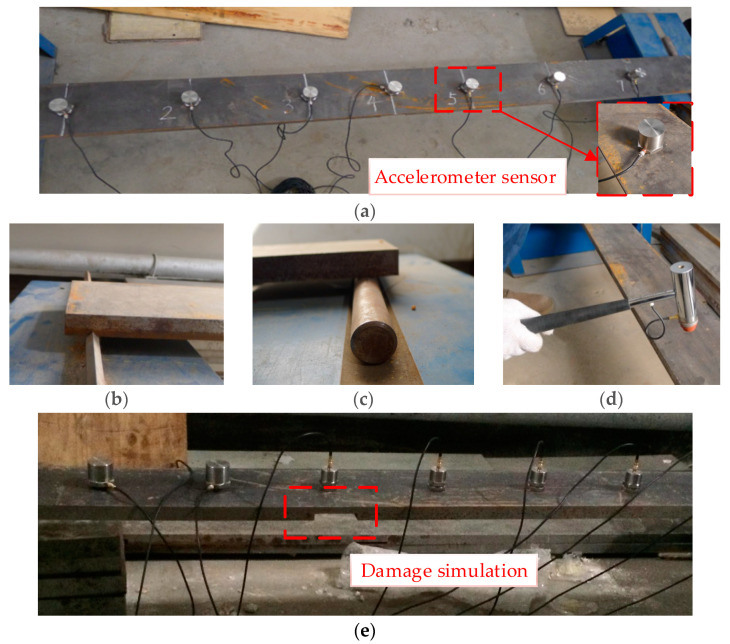
(**a**) Configuration of accelerometers on the beam, (**b**) fixed end of the beam, (**c**) roller end of the beam, (**d**) impact hammer, and (**e**) damage simulation on the beam (Case 1).

The fabricated simply supported steel beam model shown in Figure 13 was used to validate the proposed methodology. The beam material was Q235 steel with an elastic modulus of *E* = 2.06 × 10^5^ MPa. The span length, width, and height of the model beam were 1900 mm, 150 mm, and 20 mm, respectively. To simulate a simply supported beam’s supports, the procedure shown in Figure 14 was followed, in which triangle and roller joints were positioned at two sides of the beam (Figure 14a,b).

Three samples of the model beams of the same size were fabricated, with damage simulated by cross-cutting sections in two locations. Due to limitation in applied load, it is considered all damages happen in the elastic zone. The cutting location and depth are shown in Table 3. 

The DI α listed in Table 3 are calculated as follows:

(12) $$\alpha =1-\frac{\left(b{\left(h-\Delta h\right)}^{3}\right)/12}{bh^{3}/12}=1-{\left(1-\frac{\Delta h}{h}\right)}^{3}$$

where *b* and *h* are the width and depth of the beam, and Δh is the cross-cut sectional depth. 

### 4.2. Test Equipment and Laboratory Setup

The test equipment consisted of an impact hammer, acceleration sensors, and a data acquisition system. The impact hammer was an MSC-1 small-scale model with a force capacity of 0~5 kN (China Orient Institute of Noise & Vibration, Beijing, China). The integrated circuits piezoelectric (ICP) acceleration sensors used had a measurement range of 0~10 m/s^2^ with a frequency response range of 0.05 Hz~500 Hz. The data acquisition system was comprised of an INV3018A 24-bit (China Orient Institute of Noise & Vibration, Beijing, China) with eight channels. The accelerometers were directly mounted on the beams. The typical test equipment and laboratory setup are shown in Figure 14a,d.

### 4.3. Damage Identification Using the Step-by-Step Method

#### 4.3.1. Parameter Setting

**Figure 15:**
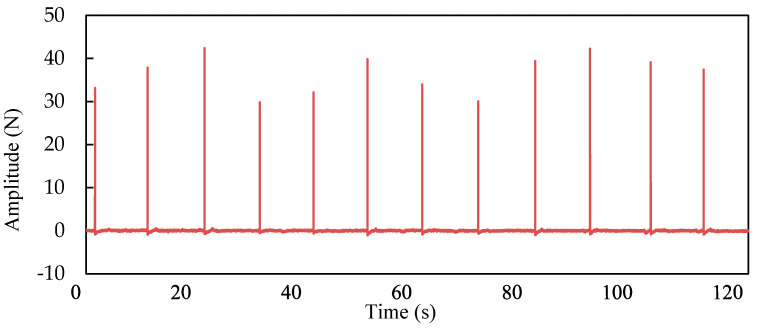
Time history of impact force.

**Figure 16:**
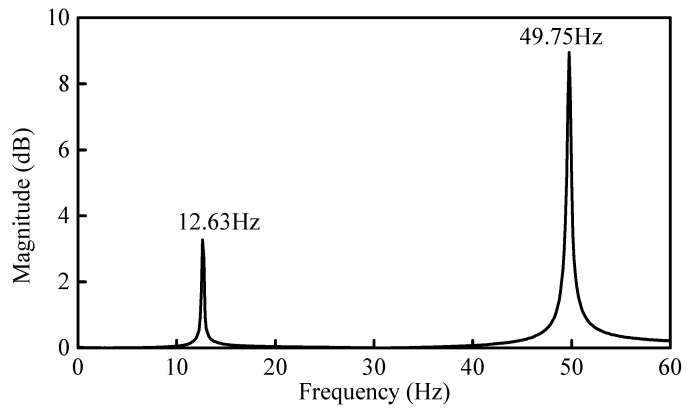
FRF spectrum of the undamaged beam in measurement Point 6.

The typical setup and arrangement of accelerometers on the beam are shown in Figure 14. The sampling frequency was 1024 Hz. The complete sampling time and frequency resolution were 120 s and 1/120 Hz, respectively. Figure 15 and Figure 16 show the time history of the applied impact load and the FRF spectrums on the beams, respectively. Based on the FRF spectrums shown in Figure 16, the first two natural frequencies of the steel beam can be determined to be 12.63 Hz and 49.75 Hz using the peak-picking method [13]. As discussed previously in Section 3.2, the section between the first-order and the second-order frequency of 15 Hz~45 Hz was selected as the analysis frequency range to calculate the CSAC indices between the measurement points on the beam. 

#### 4.3.2. Single Damage Identification (Case 1)

**Figure 17:**
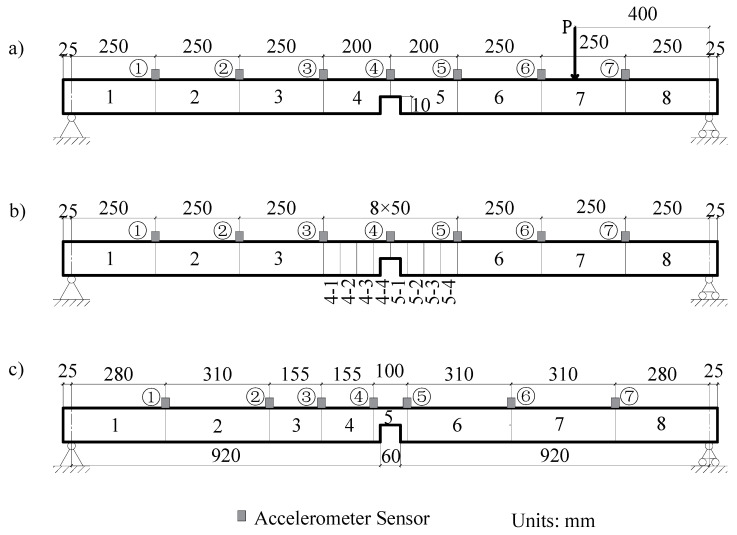
First layout of load, elements, and sensors; (**a**) with the gradual subdivision of elements as (**b**,**c**).

For the single damage Case 1 in Table 3, a 60 mm cross-cut section was produced in the middle of the beam with a depth of 10 mm. The location of damage and division of the model elements are shown in Figure 17a.

**Figure 18:**
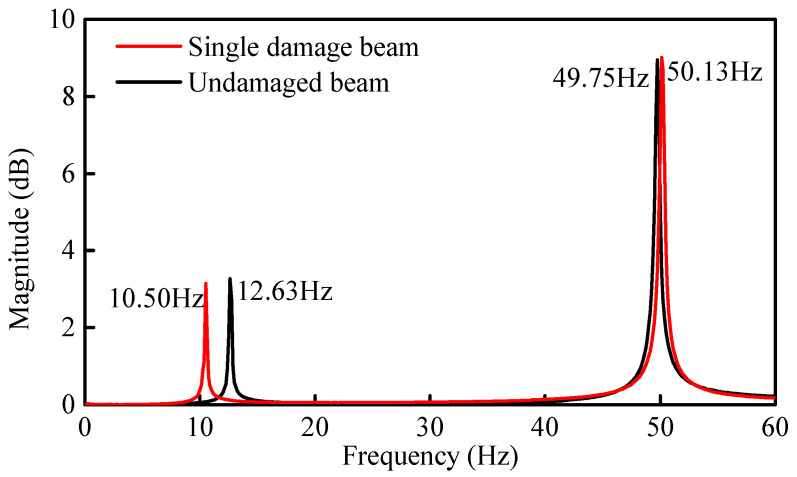
FRF spectrum of measurement Point 6 between the undamaged and damaged beam (Case 1).

Initial Identification of Damage Location: Seven sensors were arranged evenly along the span of the beam. The beam was impacted at 400 mm from the right side of the beam, i.e., roller support end (Figure 17a). The FRF spectrum of the measurement Point 6 between the undamaged and single damage beam is shown in Figure 18. The first two natural frequencies of the steel beam after damage were determined to be 10.50 Hz and 50.13 Hz, respectively.

**Figure 19:**
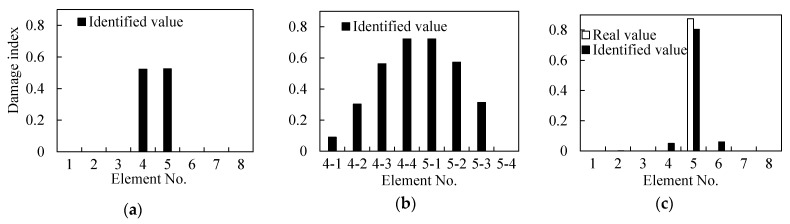
Identified values of damage (**a**) damage indices for Figure 17a, (**b**) damage indices for Figure 17b, and (**c**) damage indices for Figure 17c.

By comparing the results from initial identification, i.e., between the single damage beam (Case 1) and the undamaged beam, it was found that the first-order frequency decreases whilst the second-order frequency remained unchanged. The damage location is near the zero point of the second-order modal shape and approaches the maximum point of the first-order modal shape. This phenomenon causes the two frequencies to decay differently, i.e., a difference in the extent of reduction. The first two frequencies and six CSAC indices between the adjacent measurement points were used to construct the objective function and update the FE model. The identified damage indices of the eight elements are shown in Figure 19a. From the figure, it is seen that the damage indices of Elements 4 and 5 are 0.523 and 0.525, respectively, while the damage indices for the other elements are negligibly close to zero (0).

Refining the Damage Location: To pinpoint the location of damage further, Elements 4 and 5 were subdivided into four sub-elements each, with sequential numbering of 4-1, 4-2, 4-3, 4-4, 5-1, 5-2, 5-3, and 5-4, respectively. The subdivided FE model is shown in Figure 17b. Because other elements are undamaged, only the eight sub-elements were used as identification elements in the test. The measured impulse response and FRF data were used to update the model. The corresponding damage indices were determined as shown in Figure 19b. Evidently, the damage indices of Elements 4-4 and 5-1 are the largest in magnitude. In this step of the damage identification process, it was assumed that damage occurred on Elements 4-4 and 5-1 only, while the remaining elements were considered to be undamaged.

Quantification of Damage Severity: To confirm the damage location further, Elements 4-4 and 5-1 were merged into Element 5 and the sensors were rearranged on both sides of the element (Figure 17c). The distance between the two ends of the element with damage was 100 mm. The damage indices were determined and shown in Figure 19c. Based on this analysis, damage exists only in Element 5 whilst the other elements remained almost undamaged. This proves that the assumption that only Elements 4-4 and 5-1 were damaged is reasonable. The DI of Element 5 is 0.81, which is lower than the theoretical DI of 0.875. The assumed element length of 100 mm is larger than the actual damage length of 60 mm. This 40 mm length difference between the damaged elements partially contributed to the smaller DI than the theoretical value. Thus, if the model and the actual test errors are considered, then the preset damage would be judged to have been satisfactorily identified.

#### 4.3.3. Double Damage Identification (Case 2)

**Figure 20:**
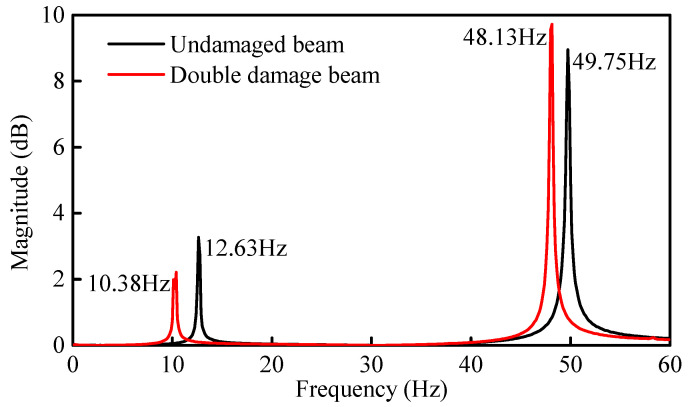
FRF spectrum of measurement Point 6 between the undamaged and damaged beam (Case 2).

**Figure 21:**
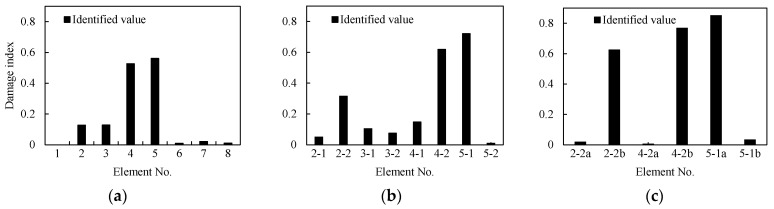
Identified values of damages in each element based on Figure 10. (**a**) damage indices of Figure 10a, (**b**) damage indices of Figure 10b, and (**c**) damage indices of Figure 10c.

Initial Identification of Damage Location: As shown in Figure 10a, two points of damage were set at the quarter span and mid-span of the beam, respectively. For this case, the theoretical damage indices of the quarter span and mid-span were 0.578 and 0.875, respectively. Based on the FRF spectrum of Point 6 in Figure 20, the first two natural frequencies of the damaged steel beam were determined to be 10.38 Hz and 48.13 Hz, respectively. Figure 21a shows the identification results of damage indices for Case 2. It can be seen from Figure 21a that the damage indices of Elements 4 and 5 have maximum values of 0.53 and 0.56, respectively. The damage indices of Elements 2 and 3 are about 0.13. The damage indices of the other elements are all close to zero (0). Therefore, it is possible to deduce that Elements 2, 3, 4, and 5 are damaged. 

Refining the Damage Location: The next step is to pinpoint and refine the damage location. As shown in Figure 21b, in order to pinpoint the damage location, the four damaged elements were subdivided into two elements. The damage indices of Elements 2-2, 4-2, and 5-1 have higher values than the other elements (Figure 10a). Furthermore, it is assumed that damage exists only in Elements 2-2, 4-2, and 5-1. The three elements were subdivided again into two elements (Figure 10c), while the remaining elements were considered as undamaged. The identified damage indices are shown in Figure 21c, in which the damage indices of Elements 2-2b, 4-2b, and 5-1a are much larger than those of the other elements. Therefore, it was concluded that damage is located in these three elements. Finally, the last step is to locate and quantify the severity of the damage, which is discussed in the subsequent text. 

**Figure 22:**
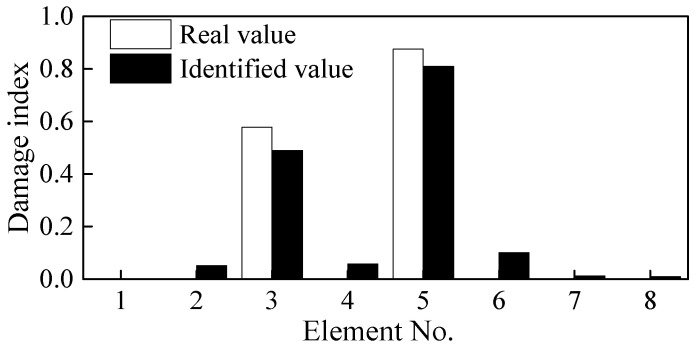
Damage indices of Figure 10d.

**Figure 23:**
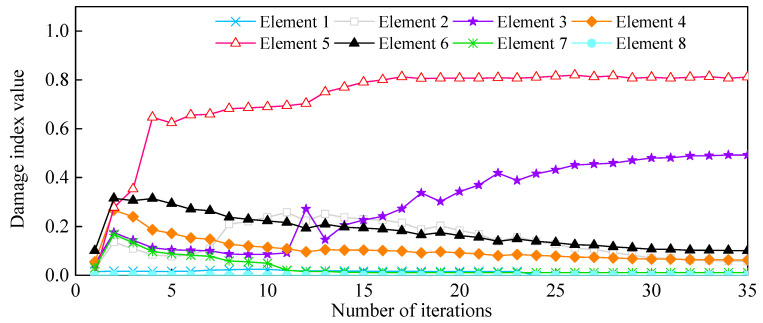
Damage indices versus the number of iterations.

Quantification of Damage Severity: As shown in Figure 10d, Elements 4-2b and 5-1a were merged into Element 5, while Element 2-2b was merged into Element 3. The measurement points on both sides of the new refined elements were accordingly rearranged. The identified damage indices of the eight elements are shown in Figure 22. The damages on Elements 3 and 5 were well defined and quantified with damage severities of 0.49 and 0.81, respectively. However, the identification damage indices are a little smaller in magnitude than the actual values when averaging them. The iterative curve of the DI in Figure 23 indicates that due to the test and model errors, the convergence efficiency of the damage indices in the experiment is slower than that of numerical simulation.

A comparison between the damage detection results of references [35,36,37,38,39] and the current method shows that the maximum difference between real and identified values for single and double damages are almost 8.2% and 15%, respectively, for the current research method. In contrast, these percentages for other studies such as Esfandiari et al. [38] with a model updating damage detection method using experimental FRF are 8% and 3.5%, respectively. This paper shows higher accuracy, however, the FRF response of the damaged structure is approximated based on the measured natural frequency of the damaged structure and the mode shapes of the intact structures that may result in a lot of errors. Sanayei et al. [39] developed a finite element model for bridges and calibrated it using strain data installed during the construction of the bridge. The developed detailed 3D finite element modeling of highway bridge was feasible with a high degree of accuracy, therefore, a tool for structural health monitoring and assistance for planning of bridge inspections, routine maintenance decisions, bridge load rating, damage assessment, and rehabilitation throughout the service life of the bridge. However, their method cannot quantify and locate damage within the bridge. Zhang et al. [35] combined the data-driven and physics-based structural health monitoring methods via physics-guided machine learning to improve the damage identification performance. This method shows better performance for severe damages with a maximum difference of real and identified values of 1% rather than a maximum difference of real and identified values for slight damages as 25%. In contrast, our proposed method is suitable for both slight and severe damages. In another study, Zhang et al. [36] studied a new method to locate multi-site structural damage using a data-driven multi-label classification (MLC) method with about 90% accuracy, which is not so practical for real bridges due to high parameters estimation in this method.

## 5. Conclusions

In this study, a new FRF-based step-by-step damage identification method is proposed to identify the damage location and quantify the severity of the damage. For method verification and effectiveness check, numerical simulation and laboratory testing on three model steel beams were conducted. The method used a cross-signature assurance criterion (CSAC) for correlating the frequency response function between the adjacent measurement points of the beams. The stiffness of the damaged beam elements is updated to converge, and the objective function is constructed by summing up the square of the CSAC index vector and the frequency residuals. The sensitivity of the CSAC index to a specific frequency range is proved through a comparison of the CSAC index difference values between the adjacent measurement points in different frequency ranges. The results showed that both the location and severity of damage on the beam are satisfactorily quantified. Overall, the main highlights from this paper’s findings are summarized as follows:

(1) When compared to traditional model updating methods, the results obtained with the proposed CSAC index exhibited satisfactory damage identification competence. The literature reviewed so far is limited in terms of integrating the CSAC index with FRF and model updating for better identification capability as demonstrated in this paper;
(2) The CSAC index in the frequency range between the first two frequencies is found to be sensitive to damage. Thus, this frequency range can be used to identify the damage location and severity in simply supported beams;
(3) The objective function can be constructed using CSAC indices of the FRFs between adjacent measurement points. The number of the constraint equations and the identification parameters can be effectively improved by increasing the number of measurement points;
(4) The damage identification method proposed in this paper exhibits insensitivity to the systematic test error. Damage, both at single and multiple locations, can be well located and quantified in the presence of noise;
(5) The step-by-step identification procedure can be used to accurately identify the damage location and determine the damage severity in simply supported beams.

## Figures and Tables

**Table 1:** Damage cases for simply supported steel beam.

Damage Cases	Damage Elements	DI
Case 1	No. 6	0.1
Case 2	No. 6	0.3
Case 3	No. 6	0.5
Case 4	No. 3, No. 6	0.2, 0.5

**Table 2:** Division of the frequency range.

Frequency Range Number	Selection of Frequency	Frequency Range (Hz)
1	Contains first two natural frequency	0~60
2	Contains only first natural frequency	0~15
3	between first and second natural frequencies	15~45
4	Contains only second natural frequency	45~60

**Table 3:** Damage simulation cases.

Damage Cases	Damage Location	Cross-Cut Sectional Depth (Δ*h)*	DI (*α*)
Case 1	middle of the beam	10 mm	0.875
Case 2	1/4, 1/2 span of the beam	5 mm, 10 mm	0.578, 0.875

## Data Availability

The data presented in this study are available on request from the corresponding author.
